# Long-term outcomes in patients with primary biliary cholangitis complicated with CREST syndrome

**DOI:** 10.1038/s41598-024-64976-8

**Published:** 2024-06-19

**Authors:** Kazumichi Abe, Manabu Hayashi, Tatsuro Sugaya, Naoto Abe, Yosuke Takahata, Masashi Fujita, Atsushi Takahashi, Kiyoshi Migita, Hiromasa Ohira

**Affiliations:** 1 Department of Gastroenterology, Fukushima Medical University School of Medicine, 1 Hikarigaoka, Fukushima City, Fukushima 960-1295 Japan; 2 Department of Rheumatology, Fukushima Medical University School of Medicine, Fukushima, Japan

**Keywords:** Primary biliary cholangitis, CREST syndrome, Prognosis, UK-PBC risk score, GLOBE score, Immunology, Gastroenterology, Rheumatology

## Abstract

Primary biliary cholangitis (PBC) is frequently associated with autoimmune disease. Although PBC complicated with CREST syndrome (PBC-CREST) has been reported, the long-term outcomes of the affected patients have not been fully investigated. Herein, the long-term outcomes of PBC-CREST were evaluated. Next, the GLOBE and UK-PBC scores were validated and compared between the PBC alone and PBC-CREST groups. A total of 302 patients who were diagnosed with PBC between December 1990 and August 2021 at Fukushima Medical University Hospital were included. The liver transplantation (LT)-free survival rates were compared between patients with PBC alone (n = 245) and those with PBC-CREST (n = 57). Moreover, 173 patients, excluding those with liver-related death/LT within 1 year after ursodeoxycholic acid administration, were divided into two subgroups (PBC alone (n = 147) and PBC-CREST (n = 26)), and the GLOBE and UK-PBC scores were compared between the subgroups. The survival rates without LT (3/5/10 years) were 92/87/80% for the PBC-alone group and 98/96/96% for the PBC-CREST group, with a significantly better prognosis in the PBC-CREST group (log-rank *P* = 0.0172). Multivariate analysis revealed that the presence of CREST syndrome is an independent protective factor for the presence of cirrhosis. The predicted 5/10/15-year risks of liver-related death or LT based on the UK-PBC score were significantly lower in the PBC-CREST group (2.4/7.6/13.2%) than in the PBC-alone group (4.8/11.8/18.8%) (*P* < 0.05). The predicted 3/5-year LT-free survival rates based on the GLOBE score were significantly higher in the PBC-CREST group (93/88%) than in the PBC-alone group (88/81%) (*P* < 0.05). Patients with PBC-CREST may have better long-term outcomes than those with PBC alone.

## Introduction

Primary biliary cholangitis (PBC) is a progressive autoimmune liver disease characterized by portal inflammation, immune-mediated destruction of the intrahepatic bile ducts, and the presence of highly specific anti-mitochondrial antibodies (AMAs) in the serum^1^. Patients with PBC occasionally experience complications from other autoimmune diseases^2–5^, such as Sjögren’s syndrome, rheumatoid arthritis, Hashimoto’s thyroiditis or scleroderma caused by either systemic sclerosis (SSc) or limited cutaneous SSc (lcSSc), a condition previously called CREST (referring to the main symptoms of calcinosis, Raynaud’s phenomenon, oesophageal dysmotility, sclerodactyly and telangiectasias) syndrome. LcSSc is accompanied by CREST symptoms, although CREST cases that involve all of these symptoms are rare, with high prevalence of Raynaud’s phenomenon, sclerodactyly and telangiectasia and lower prevalence of calcinosis and oesophageal dysmotility^6^. The association of PBC with CREST syndrome (PBC-CREST) was first described by Murray-Lyon et al. in 1970^7^. These authors reported two patients with PBC complicated with scleroderma, one of whom had CREST syndrome, and suggested that the association of PBC and CREST may be due to a common autoimmune mechanism. In 1971, Reynolds et al. reported six patients with PBC-CREST^8^. Watt et al. assessed 160 patients with PBC for the presence of additional autoimmune disease. Twelve (8%) of these patients with PBC also had scleroderma. The majority of patients (8/12, 66%) had limited disease. The CREST variant was extremely common (10/12, 83%)^9^. Although many cases have been reported since then, the aetiology and outcomes of this combined disorder remain largely unknown^10–17^.

Ursodeoxycholic acid (UDCA) is the only accepted first-line agent for the treatment of PBC^18–21^. UDCA improves liver biochemistry and slows histologic progression to cirrhosis^22,23^. Several reports have described the prognosis of PBC by several prognostic models using the Barcelona^24^, Paris I^25^, Rotterdam^26^, Toronto^27^, and Paris II^28^ criteria. However, these scoring systems have limitations due to the complexity of PBC. Recently, two newly validated scoring systems, the GLOBE score^29^ and the UK-PBC risk score^30^, were established to predict the long-term outcomes of PBC patients treated with UDCA. Several previous studies demonstrated that both the GLOBE score and the UK-PBC risk score can be used to assess risk reduction in patients who are treated with UDCA, bezafibrate (BF), fenofibrate and obeticholic acid^31–34^. However, neither of these scoring systems has been validated in patients with PBC-CREST. Some case reports suggest that patients with PBC-CREST have a better prognosis than those with PBC alone, while others report increased mortality from SSc. However, there are very few data comparing the prognosis of patients with PBC-CREST to that of patients with PBC alone. Thus, in this study, we investigated the long-term outcomes of patients with PBC-CREST and compared the GLOBE and UK-PBC scores between the PBC alone and PBC-CREST groups.

## Results

### Baseline characteristics

**Figure 1:**
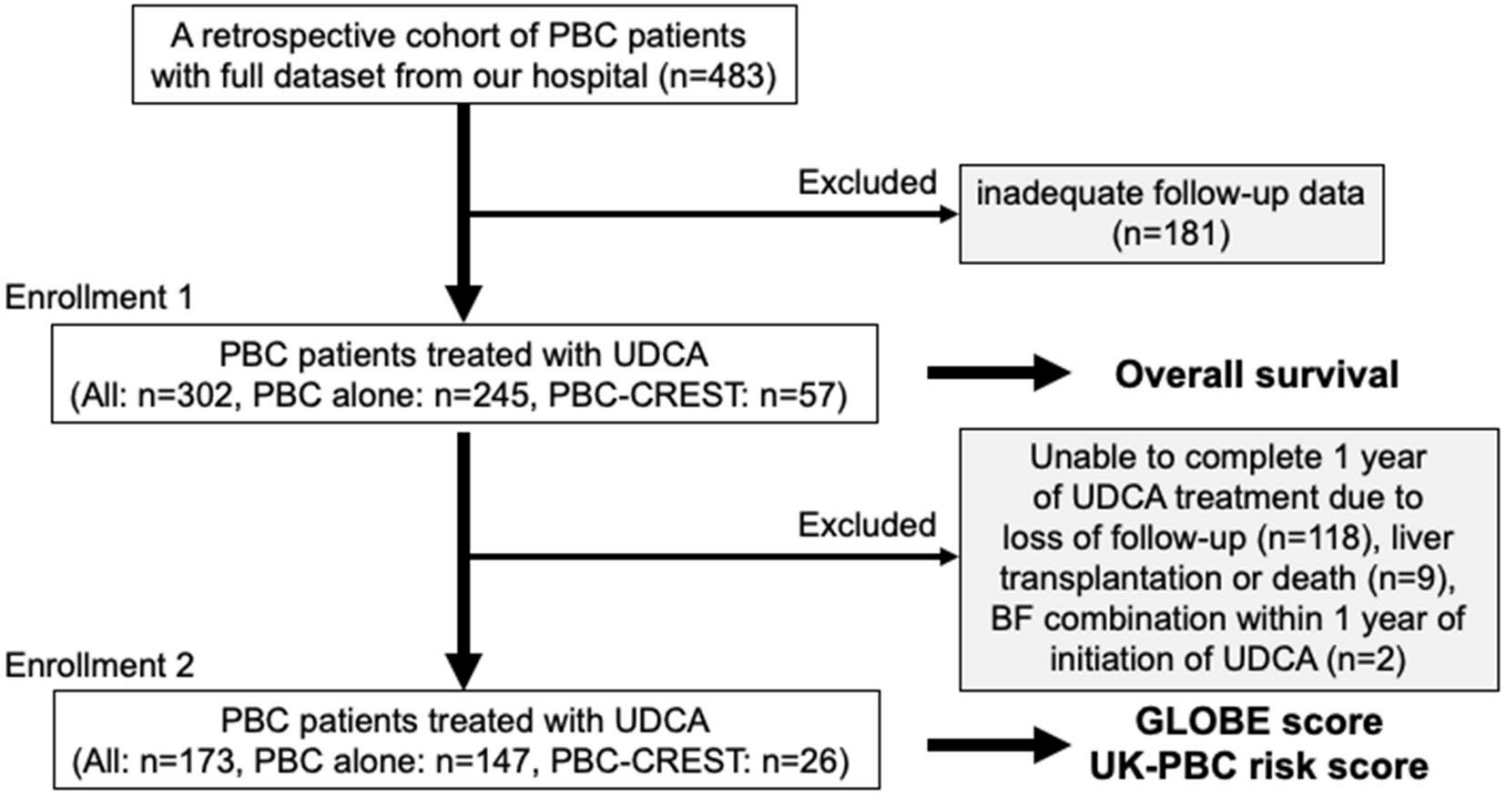
Flowchart of the patient selection process.

The clinical laboratory data from the patients with PBC-CREST are listed in Table 1. Among the 302 PBC patients, the median age at diagnosis was 59 years, and the cohort included 49 men and 253 women. Patients with PBC-CREST had significantly lower levels of alanine aminotransferase (ALT), gamma-glutamyl transpeptidase (γ-GTP), and total bilirubin (TB) than patients with PBC alone. Patients with PBC-CREST had a higher prevalence of anti-centromere antibody (ACA) positivity and a lower prevalence of AMA positivity than patients with PBC alone. Moreover, patients with PBC-CREST had a significantly lower prevalence of cirrhosis than patients with PBC alone. Although there was no significant difference in the prevalence of varices between patients with PBC-CREST and those with PBC alone, the rate of progression to cirrhosis was higher among patients with PBC alone than among patients with PBC-CREST. The median observation period was 6.3 years, and during this period, there were 6 cases of liver transplantation (LT) and 37 cases of liver-related death (LRD). Among the 245 patients with PBC alone, 207 received UDCA monotherapy, and 34 patients received combination therapy with UDCA and BF. Among the 57 patients with PBC-CREST, 56 received UDCA monotherapy, and 1 patient received combination therapy with UDCA and BF (Fig. 1).

**Table 1:** Comparison of clinical laboratory findings between patients with PBC-CREST and patients with PBC alone (N = 302).

	PBC alone	PBC-CREST	*P*
n	245	57	
Age (years), median (IQR)	59.0 (51–68)	61.5 (52–70)	0.6958
Sex (male/female)	48/197	1/56	0.0009*
Symptomatic/asymptomatic	56/189	8/49	0.1421
AST (U/L), median (IQR)	40 (26–68)	30 (22–50)	0.1551
ALT (U/L), median (IQR)	41 (25–75)	34 (22–50)	0.0376*
ALP (U/L), median (IQR)	158 (123–230)	141 (115–201)	0.1384
γ-GTP (U/L), median (IQR)	140 (71–284)	75 (44–146)	0.0002*
TB (mg/dL), median (IQR)	0.8 (0.6–1.2)	0.6 (0.5–0.9)	0.0021*
ALB (g/dL), median (IQR)	4.1 (3.6–4.3)	4.1 (3.8–4.3)	0.5802
PLT (× 10^4^/μL), median (IQR)	20.0(13.5–24.9)	20.5 (17.5–23.9)	0.5530
IgG (mg/dL), median (IQR)	1792 (1444–2189)	1640 (1420–1966)	0.1464
IgM (mg/dL), median (IQR)	321 (183–550)	336 (252–444)	0.7658
AMA-positive, n (%)	214 (87)	42 (74)	0.0097*
ACA-positive, n (%)	46 (19)	57 (100)	< 0.0001*
Scheuer stage, 1/2/3/4 (n = 159)	57/41/19/14	16/8/2/2	0.1726
Liver cirrhosis, n (%)	51 (21)	4 (7)	0.0150*
At the time of diagnosis	42	3	
During the follow up period	9	1	
Oesophagogastric varices, n (%)	46 (19)	6 (11)	0.0951
Varices without cirrhosis, n (%)	4 (1.6)	4 (7.0)	0.0226*
Hepatocellular carcinoma, n (%)	6 (2.4)	0 (0)	0.2327
Pulmonary hypertension, n (%)	1 (0.4)	1 (1.8)	0.2590
Calcinosis, n (%)	NA	14 (25)	–
Raynaud’s phenomenon, n (%)	NA	51 (89)	–
Oesophageal dysmotility, n (%)	NA	16 (28)	–
Sclerodactyly, n (%)	NA	35 (61)	–
Telangiectasia, n (%)	NA	42 (74)	–
Complete type of CREST, n (%)	NA	6 (11)	–
Observation period (years), median (IQR)	5.2 (2.4–9.9)	7.0 (3.0–10.8)	0.1284
Liver-related death, n (%)	34 (13.9)	3 (5.3)	0.0740
Liver transplantation, n (%)	6 (2.4)	0 (0)	0.2327
Liver-related death or liver transplantation, n (%)	40 (16.3)	3 (5.3)	0.0313*
SSc-related death, n (%)	NA	2 (3.5)	–
UDCA monotherapy, n (%)	207 (84.5)	56 (98.2)	0.0053*
Combination therapy with BF	34 (13.9)	1 (1.2)	0.01*

NA, not applicable. *P < 0.05. ACA, anti-centromere antibody; AMA, anti-mitochondrial antibody; BF, bezafibrate; UDCA, ursodeoxycholic acid.

### Analysis of survival after UDCA treatment

**Figure 2:**
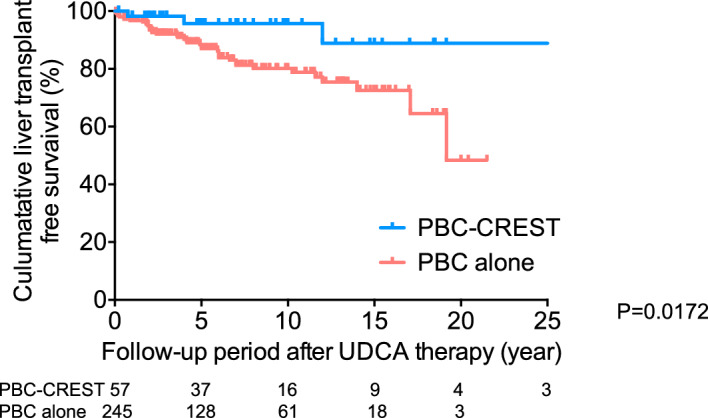
Analysis of survival after UDCA treatment (n = 302). Probability of LT-free survival in 302 patients with PBC, calculated by the Kaplan‒Meier method. Patients with PBC were classified as having PBC alone (n = 245) or PBC-CREST (n = 57) at baseline. The rate of LRD or LT was significantly lower in patients with PBC-CREST than in patients with PBC alone (log-rank test).

The median follow-up time from diagnosis was 6.3 years. The 3/5/10-year LT-free survival rates were 92/87/80% in the PBC alone group and 98/96/96% in the PBC-CREST group, with a significantly better prognosis in the PBC-CREST group (log-rank *P* = 0.0172) (Fig. 2).

### Univariate and multivariate analysis of risk and protective factors associated with liver-related death or liver transplantation in patients with primary biliary cholangitis

The results of univariate and multivariate Cox proportional hazards regression analyses of the risk factors and protective factors significantly associated with LRD/LT are shown in Supplementary Table 1 and Table 2, respectively. Univariate analysis revealed that the following factors were significantly associated with LRD/LT: ACA positivity, the presence of liver cirrhosis (LC), the presence of CREST syndrome, AST > 43 U/L, ALT > 46 U/L, ALP > 499 U/L, TB > 0.97 mg/dL, ALB ≤ 3.6 g/dL and platelet (PLT) count ≤ 12.8 × 10^4^/μL. Multivariate analysis was performed using the factors identified as significant by univariate analysis. After adjusting for confounding variables, such as age, sex, ACA status, LC status and CREST status, the hazard ratio (HR) for LRD/LT in patients with LC was 20.7 (95% confidence interval [CI] 9.46–45.3). After adjusting for confounding variables, such as age, sex, ACA status, LC status, CREST satus, AST concentration, ALT concentration and ALP concentration, the HR for LRD/LT in patients with LC was 17.7 (95% CI 7.71–40.8). After adjusting for confounding variables, such as age, sex, ACA status, LC status, CREST status, TB concentration, ALB concentration and PLT count, the HR for LRD/LT in patients with a serum ALB concentration ≤ 3.6 g/dL was 4.16 (95% CI 2.10–8.22), and the HR for LRD/LT in patients with LC was 12.9 (95% CI 5.21–32.1). The presence of LC and a serum ALB concentration ≤ 3.6 g/dL were identified as independent factors (*P* < 0.0001).

**Table 2:** Multivariate analysis of risk and protective factors associated with liver-related death or liver transplantation in patients with PBC.

	Model I		Model II		Model III	
LRD/LT vs. Non-LRD/LT	HR (95% CI)	*P*	HR (95% CI)	*P*	HR (95% CI)	*P*
Age						
≤ 58	0.92 (0.48–1.76)	0.803	0.74 (0.36–1.51)	0.417	0.89 (0.44–1.79)	0.754
> 58	1 (Ref.)		1 (Ref.)		1 (Ref.)	
Sex						
Male	1.12 (0.53–2.38)	0.765	0.89 (0.40–1.97)	0.779	1.36 (0.59–3.13)	0.464
Female	1 (Ref.)		1 (Ref.)		1 (Ref.)	
ACAs						
Positive	0.68 (0.25–1.83)	0.449	0.62 (0.22–1.76)	0.374	0.96 (0.35–2.63)	0.937
Negative	1 (Ref.)		1 (Ref.)		1 (Ref.)	
Liver cirrhosis						
Present	20.7 (9.46–45.3)	< 0.0001*	17.7 (7.71–40.8)	< 0.0001*	12.9 (5.21–32.1)	< 0.0001*
Absent	1 (Ref.)		1 (Ref.)		1 (Ref.)	
CREST syndrome						
Present	0.76 (0.17–3.33)	0.723	1.13 (0.24–5.24)	0.878	0.97 (0.22–4.21)	0.965
Absent	1 (Ref.)		1 (Ref.)		1 (Ref.)	
AST (U/L)						
> 43			2.05 (0.92–4.59)	0.079		
≤ 43			1 (Ref.)			
ALT (U/L)						
> 46			0.88 (0.41–1.93)	0.762		
≤ 46			1 (Ref.)			
ALP (U/L)						
≤ 499			1.98 (0.99–3.97)	0.051		
> 499			1 (Ref.)			
TB (mg/dL)						
> 0.97					1.67 (0.74–3.77)	0.213
≤ 0.97					1 (Ref.)	
ALB (g/dL)						
≤ 3.6					4.16 (2.10–8.22)	< 0.0001*
> 3.6					1 (Ref.)	
PLT (× 10^4^/μL)						
≤ 12.8					1.62 (0.72–3.67)	0.244
> 12.8					1 (Ref.)	

*P < 0.05 was considered significant. Model I: adjusted for age, sex, ACA status, liver cirrhosis status, and CREST status. Model II: adjusted for age, sex, ACA status, liver cirrhosis status, CREST status, AST concentration, ALT concentration, and ALP concentration. Model III: adjusted for age, sex, ACA status, liver cirrhosis status, CREST status, TB concentration, ALB concentration, and PLT count.

ACA, anti-centromere antibody; Ref., reference group; HR, hazard ratio; CI, confidence interval; LRD, liver-related death; LT, liver transplantation.

### Multivariate analysis of factors associated with liver cirrhosis in patients with primary biliary cholangitis

A total of 302 PBC patients were included in the final analysis, among whom 247 did not have LC (non-LC patients) and 55 had LC. The patients’ baseline characteristics are listed in Supplementary Table 2. Compared with PBC patients without LC, PBC patients with LC had significantly greater concentrations of AST, ALP, TB, IgG, and IgM and significantly lower ALB concentrations and PLT counts. As expected, the rate of complication with CREST syndrome was higher in PBC patients without LC than in those with LC. Multivariate analysis was performed using the factors identified as significant for LC. After adjusting for confounding variables, such as age, sex, AMA status, ACA status, and CREST status, the presence of CREST syndrome was found to be an independent protective factor. The odds ratio (OR) of LC with the presence of CREST syndrome was 0.24 (95% CI 0.07–0.83) (Table 3).

**Table 3:** Multivariate analysis of factors associated with liver cirrhosis in patients with PBC.

LC vs. Non-LC	OR (95% CI)	*P*
Age		
≤ 58	1.41 (0.78–2.57)	0.259
> 58	1 (Ref.)	
Sex		
Male	0.64 (0.26–1.55)	0.319
Female	1 (Ref.)	
AMAs		
Positive	1.78 (0.65–4.92)	0.264
Negative	1 (Ref.)	
ACAs		
Positive	1.22 (0.55–2.69)	0.622
Negative	1 (Ref.)	
CREST syndrome		
Present	0.24 (0.07–0.83)	0.024*
Absent	1 (Ref.)	

*P < 0.05 was considered significant. ACA, anti-centromere antibody; AMA, anti-mitochondrial antibody; Ref., reference group; OR, odds ratio; CI, confidence interval; LC, liver cirrhosis.

### Effects of UDCA treatment on liver biochemistry

The clinical laboratory data of the 173 patients with PBC alone or PBC-CREST at baseline, excluding patients with LRD/LT within 12 months after diagnosis and patients who were not evaluable after 12 months of UDCA administration, are presented in Supplementary Table 3. A significantly greater proportion of patients with PBC-CREST than of patients with PBC alone were women and had lower levels of γ-GTP. The effects of UDCA therapy on liver biochemistry are shown in Supplementary Fig. 1. Comparison of liver biochemistry between patients with PBC alone and patients with PBC-CREST after 12 months of treatment with UDCA showed that the TB concentration in those with PBC alone was not significantly decreased after treatment, although the ALP and ALT concentrations in patients with PBC alone were significantly decreased after treatment. On the other hand, the TB, ALP and ALT concentrations in patients with PBC-CREST were significantly decreased after treatment. The serum ALB concentration and PLT count were not significantly increased after treatment in patients with PBC alone or in those with PBC-CREST.

### Analysis of globe scores after UDCA treatment

**Figure 3:**
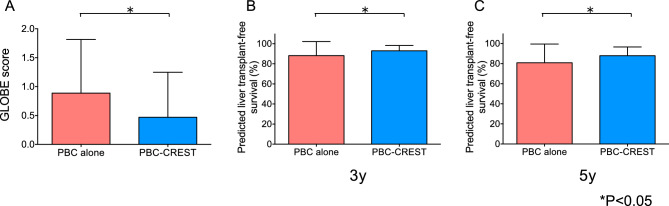
Analysis of the GLOBE score after UDCA treatment (n = 173). The GLOBE scores (**A**) and predicted 3-year (**B**) and 5-year (**C**) LT-free survival rates for patients with PBC-CREST compared to patients with PBC alone after UDCA treatment were calculated. The data are expressed as the means ± SEMs, and the statistical significance of the differences was evaluated using the nonparametric Mann‒Whitney U test.

The results of the comparison of estimated survival rates calculated using the GLOBE score between patients with PBC alone and those with PBC-CREST is shown in Fig. 3. The mean GLOBE score of the patients with PBC alone after UDCA therapy was 0.88 ± 0.93 (range: − 1.01 to 4.60), while the mean GLOBE score of those with PBC-CREST was comparatively significantly decreased (*P* < 0.05, Mann‒Whitney U test), to 0.47 ± 0.78 (range: − 1.12 to 2.09) (Fig. 3A). Moreover, 73.5% and 57.6% of patients with PBC alone and PBC-CREST, respectively, had a GLOBE score > 0.3, considered to indicate a UDCA nonresponder with a significantly shorter LT-free survival time than a matched healthy individual. In addition, the 3-/5-year LT-free survival rates calculated using the GLOBE score were significantly higher in the PBC-CREST group (93/88%) than in the PBC-alone group (88/81%) after UDCA therapy (*P* < 0.05) (Fig. 3B,C).

### analysis of UK-PBC risk scores after UDCA treatment

**Figure 4:**
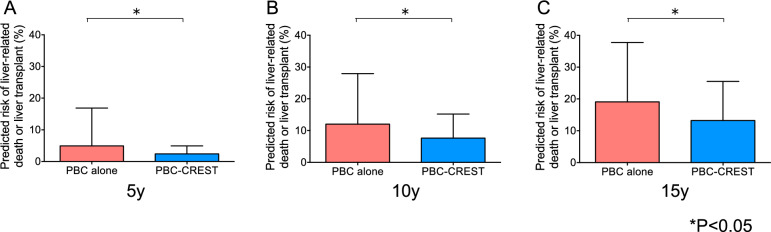
Analysis of the UK-PBC risk score after UDCA treatment (n = 173). The predicted risks of LRD or LT at 5 years (**A**), 10 years (**B**), and 15 years (**C**) were calculated 12 months after UDCA treatment and compared between patients with PBC-CREST and patients with PBC alone. The data are expressed as the means ± SEMs, and the statistical significance of the differences was evaluated using the nonparametric Mann‒Whitney U test.

The 5/10/15-year risks of LRD or LT, as predicted by the UK-PBC risk score, were significantly lower (*P* < 0.05) in patients with PBC-CREST (2.4/7.6/13.2%) than in those with PBC alone (4.8/11.8/18.8%) after UDCA therapy (Fig. 4A–C).

## Discussion

This study is the first to evaluate the long-term prognosis of patients with PBC-CREST compared with that of patients with PBC alone using the GLOBE and UK-PBC scores. In this study, the 3/5/10-year LT-free survival rates were 92/87/80% in the PBC alone group and 98/96/96% in the PBC-CREST group, with a significantly better prognosis in the PBC-CREST group. Moreover, the predicted risk of LRD and LT was significantly lower for patients with PBC-CREST than for those with PBC alone. In addition, the LT-free survival rates after UDCA therapy were significantly higher in patients with PBC-CREST. Multivariate analysis of the prevalence of cirrhosis showed that the presence of CREST syndrome was an independent protective factor. Tojo et al. reported that the survival rate 10 years after disease diagnosis was higher in patients with PBC-CREST (87.5%) than in patients with PBC alone (45.5%)^16^. Similarly, Remmel et al. reported that the survival time from PBC diagnosis was longer in anti-nuclear antibody (ANA)-positive patients than in ANA-negative patients (6.3 and 3.3 years, respectively)^35^. Moreover, ANA-negative patients more frequently had ascites and variceal haemorrhages. Other studies have demonstrated that patients with combined PBC-SSc have a significantly better outcome in terms of 10-year survival but not in terms of overall survival than those of patients with PBC alone and that the rates of jaundice progression and LT are significantly lower in patients with PBC-CREST than in patients with PBC alone^16,36^.

Recently, CREST syndrome has been considered a limited form of scleroderma, without regard to the number of symptoms present^6^. In this study, most patients with sclerodactyly and at least one other symptom were diagnosed with CREST syndrome. Patients with only Raynaud’s phenomenon and telangiectasia were included in this study because scleroderma symptoms often appear later, as described in a previous report^16^.

A higher prevalence of AMAs (63–93%) and ACAs (55–100%) has been described in several reports on PBC-CREST patients^6,10,16,36–38^. Moreover, the rate of ACA positivity differed significantly, with values of 30% in SSc patients and 70% in CREST patients. Given that ACA positivity in patients with PBC is associated with an increased risk of portal hypertension, early control of gastroesophageal varices may lead to better outcomes. Nakamura et al. reported that the rate of death from liver-associated diseases/LT was lower in ACA-positive than in ACA-negative PBC patients, although the difference was not statistically significant (log-rank *P* = 0.0510)^39^. Another study showed that the incidence of oesophageal varices was significantly higher in patients with PBC-CREST than in those with PBC alone in a cohort that excluded cirrhotic patients^16^. In this study, although the prevalence of varices did not differ significantly between patients with PBC alone and patients with PBC-CREST, among patients with varices, more patients with PBC alone than patients with PBC-CREST showed progression to cirrhosis.

Recently, two new scoring systems, the GLOBE score^29^ and the UK-PBC risk score^30^, have been developed and validated for PBC patients treated with UDCA in Western countries. The former can be used to estimate LT-free survival, while the latter can predict the risk of LRD or LT occurrence at a specific future time. The GLOBE score was established based on data collected after 12 months of UDCA treatment, but LT-free survival rates have been accurately calculated even using data collected after 2–5 years of treatment^29^. Furthermore, these scoring systems have been validated in Asian patients with PBC^40^. For these reasons, we evaluated the validity of the GLOBE and UK-PBC risk scores in our retrospective cohort. In this study, the predicted risk of LRD and LT based on the UK-PBC score was significantly lower for PBC-CREST patients than for patients with PBC alone. Moreover, the number of patients defined as UDCA nonresponsive with a GLOBE score of 0.3 or higher was significantly lower in the PBC-CREST group than in the PBC alone group. The LT-free survival rates after UDCA therapy calculated using the GLOBE score were significantly higher in PBC-CREST patients.

Despite these findings, our study has limitations. The limited sample size of this study prevents solid conclusions from being drawn, necessitating a further increase in the sample size and further experimental research.

In summary, patients with PBC-CREST manifested milder liver dysfunction associated with PBC and a lower prevalence of cirrhosis. The prevalence of ACA positivity was higher, the prevalence of AMA positivity was lower, and the prognosis was better among patients with PBC-CREST than among patients with PBC alone.

## Methods

### Study design and patient population

In this retrospective cohort study, we reviewed the clinical records of patients with PBC who were diagnosed at Fukushima Medical University Hospital (Fukushima, Japan) and related institutions between December 1990 and August 2021. The inclusion criterion was a diagnosis of PBC confirmed by liver biopsy and/or an available blood sample obtained before biopsy. Patients were diagnosed with PBC if they met at least two of the following three criteria^18^: (i) chronic elevation of the cholestatic liver enzymes ALP and γ-GTP for at least 6 months; (ii) the presence of serum AMAs detected by either indirect immunofluorescence or enzyme-linked immunosorbent assay (ELISA) using commercially available kits; and (iii) typical histological findings in biopsied liver specimens, which could be graded according to the Scheuer staging system^41^. The patients were treated with UDCA (13–15 mg/kg/day) after biopsy. The exclusion criteria included evidence of other liver diseases, such as chronic hepatitis C, chronic hepatitis B, and alcoholic liver disease (consumption of > 20 g alcohol/day). Patients who had an overlapping syndrome, systemic disorders or malignancies and were being treated with immunosuppressive therapy or chemotherapy were also excluded. LC was diagnosed based on morphological changes in the liver, such as hypertrophy of the left lateral and caudate lobes or atrophy of the right posterior hepatic lobe, identified by ultrasonography (US), computed tomography (CT), and/or magnetic resonance imaging (MRI); a finding of pseudolobule formation on histopathological examination; or the presence of portal hypertension indicated by varices and splenomegaly. Findings supporting a CREST syndrome diagnosis included the following: a finding of calcinosis on X-ray images of the extremities; a diagnosis of Raynaud’s phenomenon based on medical history; and the presence of oesophageal dysmotility shown by barium examination or endoscopy. Oesophageal dysmotility is characterized by abnormalities in oesophageal motility such as ineffective oesophageal motility, the absence of contractility, the absence of lumen-occluding peristaltic contractions, a sequence of sphincter opening followed by contraction in the oesophageal body and subsequent sphincter closing, the presence of oesophagitis/gastroesophageal reflux disease^42–44^, and sclerodactyly and telangiectasia based on objective observations^45^. The diagnosis of CREST syndrome was based on the presence of ACA positivity and at least 2 of the 5 clinical symptoms of CREST (calcinosis, Raynaud’s phenomenon, oesophageal dysmotility, sclerodactyly and telangiectasias), in accordance with a previous report^16^.

We initially enrolled 483 patients, but 181 patients were excluded because of inadequate follow-up data (Fig. 1). A total of 302 patients who underwent follow-up were included in this study. The LT-free survival rate was compared between patients with PBC alone (n = 245) and patients with PBC-CREST (n = 57). In addition, 173 of these 302 patients, excluding 9 patients with LRD/LT within 1 year after diagnosis, 118 patients who were not evaluable after 1 year of UDCA administration, and 2 patients who had concomitant use of BF within 1 year of initiation of UDCA treatment, were divided into two groups: PBC-alone (n = 147) and PBC-CREST (n = 26). The GLOBE and UK-PBC scores were compared between these groups.

### Ethical approval

The Ethics Committee of Fukushima Medical University (Fukushima Medical University protocol number: 2427) approved this study protocol, and the need for written informed consent was waived because this was an observational study. Instead of the requirement to provide written informed consent, information about this study was released, and participants were given the right to opt out. This study was conducted in accordance with the Declaration of Helsinki.

### Calculation of the globe score

The GLOBE score for each patient was calculated using the age; the concentration of bilirubin, ALP and ALB; and the PLT count^29^ days after UDCA monotherapy. The LT-free survival rates at 3, 5, 10, and 15 years were predicted using the GLOBE score^29^. The score and the prediction of LT-free survival for each patient were also calculated using data obtained 12 months after initiation of UDCA therapy.

### Calculation of the UK-PBC risk score

The UK-PBC risk score reflects the risk of LRD or LT within 5, 10, or 15 years. It is calculated using the concentrations of TB, ALP, ALT and albumin and the PLT count^30^. We determined the risk score of each patient 12 months after initiation of UDCA therapy.

### Statistical analysis

Continuous variables are reported as medians (interquartile ranges [IQRs]). Differences were compared using the Mann‒Whitney U test and Wilcoxon signed-rank test. To determine the optimal cut-off values for the factors that could distinguish between patients with LRD/LT and those without LRD/LT, receiver operating characteristic (ROC) curves were used. The cut-off values for the parameters were defined as the values closest to 100% sensitivity and specificity. Confounders with a threshold value lower than 0.05 in the univariate analysis were selected a priori based on their known associations with UDCA treatment and OS. Survival rates were calculated using the Kaplan‒Meier method, and the log-rank test was used to evaluate differences between the curves. Univariate and multivariate Cox proportional hazards regression analyses were performed to analyse the factors related to the occurrence of LRD/LT. Multivariate logistic regression analysis was performed to identify factors associated with the presence of LC. All the statistical analyses were performed using Prism 6.0 software (GraphPad Software, Inc.) and Excel Statistics (2011, Esumi Co. Ltd, Tokyo, Japan). *P* < 0.05 was considered to indicate a significant difference.

### Supplementary Information

Supplementary Figure 1.

Supplementary Tables.

## Data Availability

All the data generated or analysed during this study are included in this article. Further inquiries can be directed to the corresponding author.

## Abbreviations

ACA: Anti-centromere antibody
ALB: Albumin
ALP: Alkaline phosphatase
ALT: Alanine aminotransferase
AMA: Anti-mitochondrial antibody
ANA: Anti-nuclear antibody
AST: Aspartate aminotransferase
BF: Bezafibrate
ELISA: Enzyme-linked immunosorbent assay
γ-GTP: Gamma-glutamyl transpeptidase
H&E or HE: Haematoxylin and eosin
HBV: Hepatitis B virus
HC: Healthy control
HCV: Hepatitis C virus
HLA: Human leukocyte antigen
IQRs: Interquartile ranges
Ig: Immunoglobulin
IL: Interleukin
lcSSc: Limited cutaneous systemic sclerosis
LRD: Liver-related death
LT: Liver transplantation
PBC: Primary biliary cholangitis
PLT: Platelet
PSL: Prednisolone
PT: Prothrombin time
SSc: Systemic sclerosis
TB: Total bilirubin
UDCA: Ursodeoxycholic acid
