# Supplementary figures and images for: Long-term outcomes in patients with primary biliary cholangitis complicated with CREST syndrome
Source: Sci Rep. 2024 Jun 19;14:14124. doi: 10.1038/s41598-024-64976-8 (PMC11187228; doi:10.1038/s41598-024-64976-8)

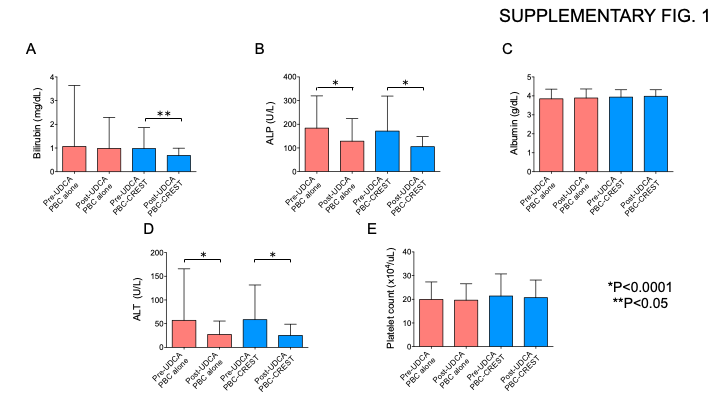

Supplement: Supplementary file 1 — Supplementary Figure 1. [file 41598_2024_64976_MOESM1_ESM.tiff]
