# Supplementary material for: Long-term outcomes in patients with primary biliary cholangitis complicated with CREST syndrome
Source: Sci Rep. 2024 Jun 19;14:14124. doi: 10.1038/s41598-024-64976-8 (PMC11187228; doi:10.1038/s41598-024-64976-8)
Supplement: Supplementary file 2 — Supplementary Tables. [file 41598_2024_64976_MOESM2_ESM.docx]

**SUPPLEMENTARY TABLE 1.** **UNIVARIATE ANALYSIS OF RISK AND PROTECTIVE FACTORS ASSOCIATED WITH LIVER-RELATED DEATH OR LIVER TRANSPLANTATION IN PATIENTS WITH PBC.**

| LRD/LT vs. Non-LRD/LT |  |  | HR (95% CI) |  | *P* |
| --- | --- | --- | --- | --- | --- |
| Age | ≤58 |  | 1.41 (0.77-2.58) |  | 0.266 |
|  | >58 |  | 1 (Ref.) |  |  |
| Sex | Male |  | 1.47 (0.70-3.09) |  | 0.304 |
|  | Female |  | 1 (Ref.) |  |  |
| AMAs | Positive |  | 3.56 (0.86-14.73) |  | 0.079 |
|  | Negative |  | 1 (Ref.) |  |  |
| ACAs | Positive |  | 0.40 (0.19-0.88) |  | 0.022^*^ |
|  | Negative |  | 1 (Ref.) |  |  |
| Liver cirrhosis | Present |  | 22.24 (10.30-48.02) |  | <0.0001^*^ |
|  | Absent |  | 1 (Ref.) |  |  |
| CREST syndrome | Present |  | 0.27（0.08-0.87） |  | 0.028^*^ |
|  | Absent |  | 1 (Ref.) |  |  |
| AST (U/L) | >43 |  | 5.83 (2.87-11.86) |  | <0.0001^*^ |
|  | ≤43 |  | 1 (Ref.) |  |  |
| ALT (U/L) | >46 |  | 2.02 (1.10-3.69) |  | 0.023^*^ |
|  | ≤46 |  | 1 (Ref.) |  |  |
| ALP (U/L) | ≤499 |  | 2.18 (1.19-3.98) |  | 0.011^*^ |
|  | >499 |  | 1 (Ref.) |  |  |
| γ-GTP (U/L) | >140 |  | 1.03 (0.56-1.89) |  | 0.913 |
|  | ≤140 |  | 1 (Ref.) |  |  |
| TB (mg/dL) | >0.97 |  | 5.44 (2.83-10.47) |  | <0.0001^*^ |
|  | ≤0.97 |  | 1 (Ref.) |  |  |
| ALB (g/dL) | ≤3.6 |  | 5.84 (3.17-10.77) |  | <0.0001^*^ |
|  | >3.6 |  | 1 (Ref.) |  |  |
| PLT (x10^4^/μL) | ≤12.8 |  | 10.42 (5.46-19.88) |  | <0.0001^*^ |
|  | >12.8 |  | 1 (Ref.) |  |  |
| IgG (mg/dL) | >1800 |  | 1.53 (0.84-2.78) |  | 0.165 |
|  | ≤1800 |  | 1 (Ref.) |  |  |
| IgM (mg/dL) | >385 |  | 1.71 (0.94-3.13) |  | 0.081 |
|  | ≤385 |  | 1 (Ref.) |  |  |

**P* < 0.05 was considered significant. Abbreviations: ACA, anticentromere antibody; AMA, antimitochondrial antibody; Ref., reference group; HR, hazard ratio; CI, confidence interval; LRD, liver-related death; LT, liver transplantation.

# SUPPLEMENTARY TABLE 2. COMPARISON OF CLINICOLABORATORY FINDINGS BETWEEN NON-LIVER CIRRHOSIS AND CIRRHOSIS IN PATIENTS WITH PBC (N=302).

|  | Non-LC | LC | P |
| --- | --- | --- | --- |
| nAge (years), median (IQR) | 247 | 55 |  |
|  | 60.0 (51-69) | 57.0 (51-67) | 0.5125 |
| Sex (male/female) | 42/205 | 7/48 | 0.4366 |
| AMA-positive, n (%) | 208 (84) | 50 (91) | 0.2029 |
| ACA-positive, n (%) | 91 (37) | 15 (27) | 0.1787 |
| CREST-present, n (%) | 53 (21) | 4 (7) | 0.0150^*^ |
| AST (U/L), median (IQR) | 35 (24-61) | 57 (41-103) | <0.0001^*^ |
| ALT (U/L), median (IQR) | 38 (25-67) | 45 (29-79) | 0.1584 |
| ALP (U/L), median (IQR) | 149 (120-208) | 187 (140-262) | 0.0213^*^ |
| γ-GTP (U/L), median (IQR) | 130 (64-253) | 111 (60-326) | 0.8550 |
| TB (mg/dL), median (IQR) | 0.7 (0.6-1.0) | 1.4 (0.8-5.6) | <0.0001^*^ |
| ALB (g/dL), median (IQR) | 4.1 (3.8-4.3) | 3.3 (2.8-3.9) | <0.0001^*^ |
| PLT (x10^4^/μL), median (IQR) | 21.6 (17.4-25.4) | 10.5 (6.1-12.8) | <00001^*^ |
| IgG (mg/dL), median (IQR) | 1678 (1384-2035) | 2056 (1711-2637) | <0.0001^*^ |
| IgM (mg/dL), median (IQR) | 312 (1175-464) | 423 (272-646) | 0.0026^*^ |

^*^P < 0.05. Abbreviations: ACA, anticentromere antibody; AMA, antimitochondrial antibody, BF, bezafibrate; UDCA, ursodeoxycholic acid.

# SUPPLEMENTARY TABLE 3. COMPARISON OF CLINICOLABORATORY FINDINGS BETWEEN PATIENTS WITH PBC-CREST AND PATIENTS WITH PBC ALONE AT BASELINE (N=173).

|  | PBC alone | PBC-CREST | P |
| --- | --- | --- | --- |
| nAge (years), median (IQR) | 147 | 26 |  |
|  | 64.0 (55-71) | 63.5 (53-70) | 0.4509 |
| Sex (male/female) | 34/113 | 1/25 | 0.0241^*^ |
| Liver cirrhosis, n (%) | 5 (3.4) | 0 (0) | 0.3399 |
| AST (U/L), median (IQR) | 39 (25-64) | 29 (22-44) | 0.1572 |
| ALT (U/L), median (IQR) | 31 (20-54) | 30 (20-52) | 0.6694 |
| ALP (U/L), median (IQR) | 148 (108-215) | 117 (102-187) | 0.3093 |
| γ-GTP (U/L), median (IQR) | 139 (72-249) | 62 (27-107) | 0.0006^*^ |
| TB (mg/dL), median (IQR) | 0.7 (0.6-1.0) | 0.7 (0.6-0.8) | 0.5765 |
| ALB (g/dL), median (IQR) | 4.0 (3.5-4.2) | 4.1 (3.8-4.2) | 0.2739 |
| PLT (x10^4^/μL), median (IQR) | 19.7 (14.4-23.9) | 20.2 (17.5-23.1) | 0.4440 |
| IgG (mg/dL), median (IQR) | 1628 (1254-1999) | 1444 (1290-1663) | 0.1457 |
| IgM (mg/dL), median (IQR) | 241 (149-415) | 234 (138-314) | 0.3644 |

**P* < 0.05 was considered significant.

**SUPPLEMENTARY FIG. 1** Effects of UDCA treatment on liver biochemistry (n = 173). The serum levels of bilirubin (A), ALP (B), albumin (C), and ALT (D) and the platelet count (E) are shown as absolute counts and were compared before UDCA treatment and after UDCA treatment for 12 months between patients with PBC alone and those with PBC-CREST. Data are the mean ± SD, and the statistical significance of the differences between the different groups was evaluated using the nonparametric Wilcoxon signed-rank test.
